# Exploring the Potential of Antidiabetic Agents as Therapeutic Approaches for Alzheimer's and Parkinson's Diseases: A Comprehensive Review

**DOI:** 10.7759/cureus.44763

**Published:** 2023-09-06

**Authors:** Mahima Koshatwar, Sourya Acharya, Roshan Prasad, Tejaswee Lohakare, Mayur Wanjari, Avinash B Taksande

**Affiliations:** 1 Department of Internal Medicine, Jawaharlal Nehru Medical College, Datta Meghe Institute of Higher Education and Research, Wardha, IND; 2 Department of Medicine, Jawaharlal Nehru Medical College, Datta Meghe Institute of Higher Education and Research, Wardha, IND; 3 Department of Child Health Nursing, Smt. Radhikabai Meghe Memorial College of Nursing, Datta Meghe Institute of Higher Education and Research, Wardha, IND; 4 Department of Research and Development, Jawaharlal Nehru Medical College, Datta Meghe Institute of Higher Education and Research, Wardha, IND; 5 Department of Physiology, Jawaharlal Nehru Medical College, Datta Meghe Institute of Higher Education and Research, Wardha, IND

**Keywords:** therapeutic approaches, neurodegenerative diseases, parkinson's disease, alzheimer's disease, antidiabetic agents

## Abstract

Alzheimer's and Parkinson's are two prevalent neurodegenerative disorders with significant societal and healthcare burdens. The search for effective therapeutic approaches to combat these diseases has led to growing interest in exploring the potential of antidiabetic agents. This comprehensive review aims to provide a detailed overview of the current literature on using antidiabetic agents as therapeutic interventions for Alzheimer's and Parkinson's diseases. We discuss the underlying pathological mechanisms of these neurodegenerative diseases, including protein misfolding, inflammation, oxidative stress, and mitochondrial dysfunction. We then delve into the potential mechanisms by which antidiabetic agents may exert neuroprotective effects, including regulation of glucose metabolism and insulin signaling, anti-inflammatory effects, modulation of oxidative stress, and improvement of mitochondrial function and bioenergetics. We highlight in vitro, animal, and clinical studies that support the potential benefits of antidiabetic agents in reducing disease pathology and improving clinical outcomes. However, we also acknowledge these agents' limitations, variability in treatment response, and potential side effects. Furthermore, we explore emerging therapeutic targets and novel approaches, such as glucagon-like peptide-1 receptor (GLP-1R) agonists, insulin sensitizer drugs, neuroinflammation-targeted therapies, and precision medicine approaches. The review concludes by emphasizing the need for further research, including large-scale clinical trials, to validate the efficacy and safety of antidiabetic agents in treating Alzheimer's and Parkinson's disease. The collaboration between researchers, clinicians, and pharmaceutical companies is essential in advancing the field and effectively treating patients affected by these debilitating neurodegenerative disorders.

## Introduction and background

Alzheimer's and Parkinson's diseases are two prevalent neurodegenerative disorders that pose significant global challenges to public health [1]. Alzheimer's disease is characterized by progressive cognitive decline, memory loss, and behavioral changes. It is the most common cause of dementia, affecting millions globally. The pathological hallmarks of Alzheimer's disease include the accumulation of amyloid-beta plaques and neurofibrillary tangles in the brain, leading to synaptic dysfunction and neuronal loss [2]. Parkinson's disease, on the other hand, primarily manifests as motor symptoms such as tremors, rigidity, and bradykinesia. It is caused by the degeneration of dopaminergic neurons in the substantia nigra region of the brain. Parkinson's disease is associated with nonmotor symptoms such as cognitive impairment, depression, and autonomic dysfunction [3].

Currently, the available treatments for Alzheimer's and Parkinson's diseases aim to alleviate symptoms and slow the progression of the diseases. In Alzheimer's disease, cholinesterase inhibitors (e.g., donepezil and rivastigmine) and the N-methyl-D-aspartate (NMDA) receptor antagonist memantine are commonly prescribed to enhance cognitive function. However, these medications have limited efficacy and do not provide a cure for the disease [4]. In Parkinson's disease, the gold standard treatment is levodopa, which helps replenish dopamine levels in the brain. Other medications, such as dopamine agonists, catechol-O-methyltransferase (COMT), and monoamine oxidase type B (MAO-B) inhibitors, are also used to manage symptoms. However, long-term use of levodopa can lead to motor complications and dyskinesias [5].

Growing evidence suggests a potential link between diabetes and neurodegenerative diseases like Alzheimer's and Parkinson's. Epidemiological studies have revealed that individuals with diabetes have an increased risk of developing dementia, including Alzheimer's disease. Similarly, diabetes has been associated with a higher incidence of Parkinson's disease [6].

Mechanisms proposed to explain the link between diabetes and neurodegeneration include insulin resistance, impaired glucose metabolism, chronic inflammation, oxidative stress, and mitochondrial dysfunction. These shared pathological processes could contribute to the development and progression of diabetes and neurodegenerative diseases [7]. Given the potential connection between diabetes and neurodegenerative disorders, exploring the role of antidiabetic agents as therapeutic approaches for Alzheimer's and Parkinson's disease holds promise. This comprehensive review examines the current evidence and sheds light on the potential benefits and challenges of utilizing antidiabetic agents to manage these neurodegenerative conditions.

## Review

Methodology

The methodology involved a comprehensive literature search strategy using multiple electronic databases, including PubMed, Scopus, and Google Scholar. The search terms used were related to "antidiabetic agents," "Alzheimer's disease," "Parkinson's disease," "neurodegenerative diseases," and "therapeutic approaches." In addition to electronic database searches, the reference lists of relevant articles and review papers were manually searched for additional studies. No language restrictions were applied, but only studies published up to the current knowledge as of 2023 were included. The inclusion criteria were defined to select studies that were relevant and of high quality. Studies investigating the potential of antidiabetic agents as therapeutic approaches for Alzheimer's and Parkinson's diseases were included. This encompassed in vitro studies, animal models, and clinical studies of various designs. Exclusion criteria included studies focusing solely on diabetes management unrelated to neurodegenerative diseases and review articles, editorials, commentaries, and conference abstracts. Two independent reviewers screened the titles and abstracts, followed by a full-text assessment of selected articles. Disagreements were resolved through consensus or consultation with a third reviewer if needed. The methodology aimed to include high-quality studies that contributed to a comprehensive understanding of the potential of antidiabetic agents as therapeutic approaches for Alzheimer's and Parkinson's disease. By employing a rigorous search strategy and applying strict inclusion and exclusion criteria, a robust selection of studies was identified to inform the review.

Understanding the pathogenesis of Alzheimer's and Parkinson's diseases

A Brief Explanation of the Key Mechanisms Involved in Alzheimer's Disease

Alzheimer's is a complex neurodegenerative disorder characterized by the accumulation of abnormal protein aggregates in the brain, contributing to the progressive decline in cognitive function. The two primary pathological hallmarks of Alzheimer's disease are amyloid-beta plaques and neurofibrillary tangles.

Amyloid-beta plaques: Amyloid-beta is a peptide derived from the amyloid precursor protein (APP). In Alzheimer's disease, there is an abnormal processing of APP, resulting in the accumulation of amyloid-beta peptides. These peptides have a propensity to aggregate, leading to the formation of insoluble plaques within the brain. Amyloid-beta plaques disrupt the normal functioning of neurons by impairing synaptic communication, inducing oxidative stress, and triggering inflammatory responses. The presence of amyloid-beta plaques is an early pathological event in Alzheimer's disease [8,9].

Neurofibrillary tangles: Neurofibrillary tangles are intracellular aggregates formed by the hyperphosphorylation and subsequent aggregation of the tau protein. Tau protein is essential for stabilizing microtubules, which are critical for maintaining the structural integrity and proper functioning of neurons. In Alzheimer's disease, abnormal modifications of tau protein led to its misfolding and aggregation into insoluble tangles. The accumulation of neurofibrillary tangles disrupts the normal cytoskeletal structure of neurons, impairs axonal transport, and ultimately leads to neuronal dysfunction and cell death. Neurofibrillary tangles are typically observed in the later stages of the disease and correlate strongly with cognitive decline [10,11].

The presence of amyloid-beta plaques and neurofibrillary tangles in the brains of individuals with Alzheimer's disease contributes to the progressive neurodegeneration and cognitive impairment associated with the disease. Understanding the underlying mechanisms of these pathological features is crucial for developing effective therapeutic approaches aimed at targeting and modifying the progression of Alzheimer's disease [12].

A Brief Explanation of the Key Mechanisms Involved in Parkinson's Disease

Parkinson's disease is a neurodegenerative disorder characterized by the selective degeneration of dopaminergic neurons in the substantia nigra region of the brain. This degeneration leads to a deficiency of dopamine, a neurotransmitter involved in motor control and coordination. The loss of dopamine-producing neurons is responsible for the characteristic motor symptoms observed in Parkinson's disease, such as tremors, rigidity, bradykinesia (slowness of movement), and postural instability [13].

The pathogenesis of Parkinson's disease involves several key mechanisms.

Alpha-synuclein accumulation: Parkinson's disease is closely associated with the accumulation of abnormal protein aggregates composed mainly of alpha-synuclein. These aggregates, known as Lewy bodies, disrupt normal cellular function and contribute to the degeneration of dopaminergic neurons. The exact mechanisms by which alpha-synuclein aggregates contribute to neurodegeneration are still under investigation, but their presence is a hallmark of Parkinson's disease pathology [14,15].

Mitochondrial dysfunction: Dysfunction in mitochondrial energy metabolism and impaired mitochondrial quality control mechanisms have been implicated in the pathogenesis of Parkinson's disease. Mitochondria are crucial in generating cellular energy (adenosine triphosphate or ATP) through oxidative phosphorylation. In Parkinson's disease, there is evidence of mitochondrial dysfunction, including decreased ATP production, increased oxidative stress, and impaired clearance of damaged mitochondria. These mitochondrial abnormalities contribute to neuronal degeneration and cell death in the substantia nigra [16,17].

The interplay between alpha-synuclein accumulation and mitochondrial dysfunction further exacerbates the neurodegenerative process in Parkinson's disease. Oxidative stress resulting from mitochondrial dysfunction can promote alpha-synuclein aggregation, while alpha-synuclein aggregates can impair mitochondrial function and exacerbate oxidative stress. This vicious cycle contributes to the progressive loss of dopaminergic neurons and the clinical manifestations of Parkinson's disease [18,19]. Understanding the underlying mechanisms involved in the selective degeneration of dopaminergic neurons is essential for developing targeted therapeutic strategies to slow disease progression, preserve dopamine function, and alleviate symptoms of Parkinson's disease.

Shared Pathological Features and Overlapping Molecular Pathways

Protein misfolding and aggregation: Both diseases involve the abnormal accumulation and aggregation of specific proteins. In Alzheimer's disease, amyloid-beta peptides aggregate to form plaques, while in Parkinson's disease, alpha-synuclein forms Lewy bodies. Protein misfolding and aggregation contribute to neuronal toxicity, disrupt cellular processes, and ultimately lead to neurodegeneration [20,21].

Inflammation: Chronic inflammation is a prominent feature observed in both Alzheimer's and Parkinson's diseases. Activated microglia, the brain's immune cells, release pro-inflammatory cytokines and contribute to neuroinflammation. This sustained inflammatory response exacerbates neuronal damage and contributes to disease progression in both conditions [22,23].

Oxidative stress: Increased production of reactive oxygen species (ROS) and impaired antioxidant defense mechanisms contribute to oxidative stress, a common feature in both diseases. Oxidative stress leads to cellular damage, including lipid peroxidation, DNA damage, and protein oxidation. It plays a significant role in the neurodegenerative process of Alzheimer's and Parkinson's diseases [24,25].

Impaired protein clearance: Both diseases disrupt protein clearance mechanisms, such as autophagy and the ubiquitin-proteasome system. Impaired protein clearance accumulates toxic protein aggregates, further contributing to neuronal dysfunction and disease progression [26,27].

Understanding the shared pathological features and molecular pathways between Alzheimer's and Parkinson's diseases provides valuable insights into potential therapeutic targets relevant to both conditions. By targeting these common mechanisms, such as protein misfolding, inflammation, oxidative stress, and impaired protein clearance, interventions aimed at modulating these pathways may hold promise for developing effective treatments for both Alzheimer's and Parkinson's diseases. The subsequent sections of this review will delve into the potential of antidiabetic agents as therapeutic approaches, considering their effects on these shared mechanisms.

Antidiabetic agents as potential therapeutic approaches

Overview of Commonly Used Antidiabetic Agents and Their Mechanisms of Action

Antidiabetic agents comprise a diverse group of medications utilized to manage diabetes, aiming to regulate blood glucose levels. These agents target various aspects of glucose metabolism and insulin signaling, offering different mechanisms of action. Here are some commonly used antidiabetic agents.

Metformin: Metformin, a widely prescribed biguanide, is a first-line medication for type 2 diabetes. It primarily works by reducing hepatic glucose production and enhancing peripheral glucose uptake. Metformin also improves insulin sensitivity in target tissues, such as skeletal muscle and adipose tissue, without promoting excessive insulin secretion [28,29].

Sulfonylureas: Sulfonylureas, including glipizide and glyburide, stimulate insulin secretion from pancreatic beta cells by binding to ATP-sensitive potassium channels. By closing these channels, sulfonylureas lead to membrane depolarization and subsequent calcium influx, triggering insulin release [30,31].

Thiazolidinediones: Thiazolidinediones, such as pioglitazone, improve insulin sensitivity by activating peroxisome proliferator-activated receptor gamma (PPARγ). PPARγ is a nuclear receptor involved in regulating glucose and lipid metabolism. Thiazolidinediones promote adipocyte differentiation, enhance glucose uptake in peripheral tissues, and reduce insulin resistance [32,33].

Dipeptidyl peptidase-4 (DPP-4) inhibitors: DPP-4 inhibitors, like sitagliptin and saxagliptin, prolong the action of incretin hormones, particularly glucagon-like peptide-1 (GLP-1). GLP-1 and other incretin hormones stimulate glucose-dependent insulin secretion and inhibit glucagon release. DPP-4 inhibitors inhibit the enzymatic degradation of incretin hormones, thereby enhancing their activity and promoting better glycemic control [34,35].

These antidiabetic agents represent a selection of commonly used medications with distinct mechanisms of action to manage diabetes and regulate blood glucose levels. The subsequent sections of this review will explore the potential neuroprotective effects of these antidiabetic agents and their implications for Alzheimer's and Parkinson's disease.

Evidence of the neuroprotective effects of antidiabetic agents

In Vitro Studies

In vitro studies have provided valuable insights into the potential neuroprotective effects of antidiabetic agents against the pathological features associated with Alzheimer's and Parkinson's diseases. These studies have highlighted the following findings.

Metformin: In Alzheimer's disease models, metformin has demonstrated neuroprotective effects. It has been shown to reduce amyloid-beta production, which is responsible for forming plaques, and inhibit tau protein phosphorylation, which is associated with the formation of neurofibrillary tangles. Additionally, metformin exhibits anti-inflammatory properties, reducing neuroinflammation and its detrimental effects on neuronal function. These findings suggest that metformin can potentially mitigate Alzheimer's disease-related pathology [36,37].

Thiazolidinediones: Thiazolidinediones have shown neuroprotective effects in Alzheimer's disease models. They have been found to promote neurogenesis, the generation of new neurons, which is important for neuronal repair and regeneration. Thiazolidinediones also exhibit anti-inflammatory properties, reducing neuroinflammatory responses. Furthermore, they have been shown to attenuate tau pathology, potentially preventing the formation of neurofibrillary tangles. These findings suggest that thiazolidinediones may have therapeutic potential in Alzheimer's disease by targeting multiple pathological mechanisms [38,39].

DPP-4 inhibitors: In vitro studies have indicated that DPP-4 inhibitors possess neuroprotective properties in Alzheimer's disease models. They have been shown to protect against neuronal death and reduce neuroinflammation, thereby preserving neuronal function. Additionally, DPP-4 inhibitors have been found to enhance cognitive function, potentially through their effects on neurotransmitter systems. These findings suggest that DPP-4 inhibitors may offer neuroprotective benefits and improve cognitive outcomes in Alzheimer's disease [40,41].

These in vitro studies provide valuable preliminary evidence supporting the neuroprotective potential of antidiabetic agents in the context of Alzheimer's and Parkinson's diseases. However, it is important to note that further investigations, including preclinical and clinical studies, are needed to validate these findings and establish the efficacy and safety of these agents in treating neurodegenerative diseases [42-45].

Animal Models

Metformin: Animal models of Parkinson's disease have demonstrated the beneficial effects of metformin. Metformin has been shown to improve motor function, including motor coordination and balance. It has also been found to protect against dopaminergic neuronal loss, which is a hallmark of Parkinson's disease. Additionally, metformin exhibits anti-inflammatory properties, reducing neuroinflammation and its detrimental effects on neuronal function. These findings suggest that metformin holds promise as a neuroprotective agent in Parkinson's disease [46,47].

Thiazolidinediones: Animal models of Parkinson's disease have also revealed the neuroprotective effects of thiazolidinediones. Thiazolidinediones have been found to preserve dopaminergic neurons, the cells primarily affected in Parkinson's disease. They attenuate neuroinflammation, reducing the inflammatory responses that contribute to neurodegeneration. Moreover, thiazolidinediones have improved motor symptoms associated with Parkinson's disease, suggesting their potential as therapeutic agents for symptom management [48,49].

These animal studies provide valuable insights into the neuroprotective effects of antidiabetic agents, specifically metformin and thiazolidinediones, in the context of Parkinson's disease. However, further research, including clinical trials, is necessary to confirm these findings and determine the optimal use of these agents in treating Parkinson's disease in humans.

Clinical studies investigating the use of antidiabetic agents in neurodegenerative diseases

Alzheimer's Disease

Enhanced cognitive function: Specific antidiabetic agents have demonstrated the potential to enhance cognitive function among individuals diagnosed with Alzheimer's. These enhancements encompass memory retention, attention span, and executive functionality. Noteworthy examples include studies indicating that the administration of metformin or thiazolidinediones correlates with improved cognitive performance and decelerated cognitive deterioration in Alzheimer's patients. Nevertheless, it is essential to acknowledge that the cognitive advantages illustrated above have not been universally replicated across all investigations [50-53]. Further investigation is warranted to elucidate the factors contributing to the variable outcomes observed.

Reduced disease progression: The potential benefits of antidiabetic agents in slowing the progression of Alzheimer's disease. These include a decrease in the rate of cognitive decline and a reduction in the accumulation of amyloid-beta plaques, a key pathological feature of the disease. However, other studies have not found significant effects on disease progression [54,55].

Divergent impact on different populations: The collective findings of clinical investigations exploring the utilization of antidiabetic agents in the context of Alzheimer's disease have exhibited a heterogeneous nature. While certain studies have demonstrated favorable results, contrasting outcomes marked by the absence of noteworthy effects or conflicting findings have also been reported. These inconsistencies could potentially be elucidated by disparities in multiple factors, including study architecture, specific patient demographics, variations in drug protocols, and the diverse array of metrics employed to gauge outcomes [56-62].

To better understand the potential benefits of antidiabetic agents in Alzheimer's disease treatment, ongoing clinical trials are being conducted. These trials aim to investigate further the efficacy and safety of various antidiabetic agents, including metformin, thiazolidinediones, and DPP-4 inhibitors, in individuals with Alzheimer's. The results of these trials will provide valuable insights into the role of antidiabetic agents as therapeutic approaches for Alzheimer's disease and may guide future treatment strategies.

Parkinson's Disease

Improved motor symptoms: Some preliminary clinical studies have indicated the potential benefits of antidiabetic agents in improving motor symptoms in Parkinson's disease. These benefits include reduced motor fluctuations, improved motor function, and increased quality of life. For example, metformin has been associated with improved motor function and reduced levodopa-induced dyskinesias in small-scale clinical studies. However, more robust clinical trials are needed to confirm these findings [63,64].

Reduced disease progression: Evidence suggests that antidiabetic agents may modify Parkinson's disease, potentially slowing disease progression. Clinical studies have reported slower rates of disease progression, as measured by the Unified Parkinson's Disease Rating Scale (UPDRS) and other clinical assessments, in patients treated with certain antidiabetic agents. These findings warrant further investigation in larger clinical trials [65,66].

It is important to note that the clinical studies investigating the use of antidiabetic agents in Parkinson's disease are relatively limited in size and scope. Therefore, more extensive and well-designed clinical trials are needed to establish antidiabetic agents' efficacy, optimal dosing, and long-term safety in Parkinson's disease treatment.

Mechanisms underlying the neuroprotective effects of antidiabetic agents

Regulation of Glucose Metabolism and Insulin Signaling

Enhanced glucose uptake: Antidiabetic agents, such as metformin, can enhance glucose uptake in neurons by activating adenosine monophosphate-activated protein kinase (AMPK). AMPK activation leads to increased translocation of glucose transporter proteins, such as glucose transporter 4 (GLUT4), to the neuronal cell membrane, facilitating glucose uptake from the extracellular space. This increased glucose availability provides neurons with a more efficient energy source, supporting their metabolic needs and overall function. Improved glucose utilization can enhance neuronal viability and resilience against neurodegenerative insults [67,68].

Insulin signaling modulation: Antidiabetic agents can also modulate insulin signaling pathways in the brain, thereby exerting neuroprotective effects. Insulin receptors are expressed in various brain regions, including areas affected by neurodegenerative diseases. Antidiabetic agents may enhance insulin signaling by promoting receptor activation, downstream signaling cascades, and transcriptional regulation. Activation of insulin signaling pathways in neurons can have several beneficial effects [69-71].

First, it can enhance neurotrophic support by stimulating the release of neurotrophic factors, such as brain-derived neurotrophic factor (BDNF), which promotes neuronal survival, growth, and synaptic plasticity. Neurotrophic factors play critical roles in maintaining neuronal integrity and function, and their dysregulation is implicated in neurodegenerative diseases [72]. Second, insulin signaling modulation can regulate neuronal metabolism and energy homeostasis. It influences neurons' glucose uptake, glycolysis, mitochondrial function, and lipid metabolism. By promoting efficient utilization of glucose and other energy substrates, antidiabetic agents can support optimal neuronal metabolism and mitigate energy deficits that contribute to neurodegeneration [73]. Furthermore, activation of insulin signaling pathways can enhance cell survival mechanisms and protect neurons from apoptotic and oxidative stress-induced cell death. Insulin signaling can activate prosurvival signaling pathways, such as the phosphoinositide 3-kinase (PI3K)/Akt pathway, which promotes cell survival and inhibits proapoptotic pathways [74].

Anti-inflammatory Effects

Chronic inflammation is a key contributor to the development and progression of neurodegenerative diseases. In these conditions, inflammatory processes in the central nervous system, including the activation of microglia and the release of pro-inflammatory cytokines, contribute to neuronal damage and loss. Antidiabetic agents have demonstrated anti-inflammatory properties that can help attenuate neuroinflammation and protect against neurodegeneration [75].

Inhibition of pro-inflammatory cytokines: Among the antidiabetic agents, particularly thiazolidinediones, a capacity exists to inhibit the synthesis of pro-inflammatory cytokines. Notably, tumor necrosis factor-alpha (TNF-α) and interleukin-6 (IL-6) are included in these cytokines. These molecules play a pivotal role as mediators of inflammation and have also been associated with processes related to neurodegeneration. By exerting control over their production, antidiabetic agents exhibit the potential to curtail the inflammatory response witnessed within the brain, ultimately leading to a reduction in neuronal damage [76]

Modulation of microglial activation: Microglia, the resident immune cells of the central nervous system, play a crucial role in neuroinflammation. Microglia can become activated in response to pathological stimuli and release pro-inflammatory factors. Antidiabetic agents have been found to modulate microglial activation and polarization, leading to a shift from a pro-inflammatory state to an anti-inflammatory or neuroprotective phenotype. This modulation of microglial function helps dampen the inflammatory response and create a more favorable environment for neuronal survival [77].

Antidiabetic agents can help reduce the detrimental effects of chronic inflammation in neurodegenerative diseases by targeting these inflammatory mechanisms. The inhibition of pro-inflammatory cytokines and modulation of microglial activation contributes to the neuroprotective effects of these agents, promoting a more balanced immune response and potentially slowing down disease progression. Understanding these mechanisms is crucial for developing targeted therapeutic strategies that address the inflammatory component of neurodegenerative diseases.

Oxidative Stress Modulation

Oxidative stress, a key contributor to neurodegeneration, arises from an imbalance between the production of ROS and the antioxidant defense systems in the body. Antidiabetic agents have shown the ability to modulate oxidative stress and protect neurons from oxidative damage, potentially mitigating neurodegenerative processes [78].

One mechanism by which antidiabetic agents exert their antioxidative effects is through ROS scavenging. Certain agents, such as metformin and thiazolidinediones, possess direct antioxidant properties, enabling them to neutralize ROS directly and reduce oxidative stress in neurons. Acting as free radical scavengers, these agents help maintain redox balance and protect cellular components from oxidative damage [79].

In addition to ROS scavenging, antidiabetic agents may induce the expression of endogenous antioxidant defense mechanisms. This includes upregulating the production of key antioxidant enzymes, such as superoxide dismutase (SOD) and glutathione peroxidase (GPx). SOD catalyzes the conversion of superoxide radicals into hydrogen peroxide, while GPx aids in breaking hydrogen peroxide into harmless molecules. By enhancing the expression and activity of these enzymes, antidiabetic agents bolster the cellular antioxidant defense system, reducing oxidative stress-induced damage [80].

By modulating oxidative stress and promoting a favorable redox environment, antidiabetic agents have the potential to counteract neurodegenerative processes associated with conditions like Alzheimer's and Parkinson's diseases. These mechanisms highlight the multifaceted neuroprotective properties of antidiabetic agents and further support their potential as therapeutic approaches in managing neurodegenerative diseases.

Mitochondrial Function and Bioenergetics

Impaired mitochondrial function and energy metabolism are recognized as key factors in the development and progression of neurodegenerative diseases such as Alzheimer's and Parkinson's diseases. Mitochondria are crucial in cellular energy production, calcium homeostasis regulation, and redox balance maintenance. Dysfunctional mitochondria can lead to energy deficits, increased ROS production, and impaired cellular function [81].

Antidiabetic agents have shown the ability to influence mitochondrial function and bioenergetics, promoting neuronal health and potentially exerting neuroprotective effects. One mechanism by which antidiabetic agents can impact mitochondria is by promoting mitochondrial biogenesis. For instance, thiazolidinediones have been shown to activate peroxisome proliferator-activated receptor gamma coactivator-1 alpha (PGC-1α), a key regulator of mitochondrial biogenesis. This activation increases the number and functional capacity of mitochondria in neurons, enhancing energy production and cellular function [82].

In addition to promoting mitochondrial biogenesis, antidiabetic agents can improve cellular bioenergetics. They can enhance mitochondrial respiration, ATP production, and metabolic flexibility, essential for neuronal function. By optimizing mitochondrial function, antidiabetic agents help provide an adequate energy supply to neurons, maintaining their vitality and supporting their survival. This improvement in bioenergetics can help protect against neurodegeneration by reducing energy deficits and enhancing cellular resilience [83].

The neuroprotective effects of antidiabetic agents in neurodegenerative diseases are multifaceted and can be attributed to their regulation of various mechanisms. Antidiabetic agents help optimize energy utilization in the brain by targeting glucose metabolism and insulin signaling. The anti-inflammatory properties of these agents contribute to the mitigation of neuroinflammation, which is a key driver of neurodegeneration. Antioxidant effects reduce oxidative stress and limit cellular damage caused by ROS. Moreover, the modulation of mitochondrial function and bioenergetics ensures an efficient energy supply for neuronal processes and promotes cellular health [84].

Regulating glucose metabolism, insulin signaling, anti-inflammatory properties, modulation of oxidative stress, and improvement of mitochondrial function and bioenergetics contribute to the potential therapeutic benefits of antidiabetic agents in neurodegenerative diseases such as Alzheimer's and Parkinson's disease. However, further research is needed to fully understand the underlying mechanisms and optimize the use of these agents in the clinical setting.

Potential challenges and future directions

Limitations and Side Effects of Antidiabetic Agents

Drug-specific side effects: Each antidiabetic agent has its profile of side effects that must be considered. For example, metformin may cause gastrointestinal disturbances such as diarrhea or nausea. Sulfonylureas can increase the risk of hypoglycemia, while thiazolidinediones may be associated with weight gain and an increased risk of cardiovascular events. It is important to evaluate individual patient profiles carefully, considering their medical history, comorbidities, and potential drug interactions, to choose the most suitable antidiabetic agent and monitor for adverse effects [85].

Variability in treatment response: Individuals' responses to antidiabetic agents in neurodegenerative diseases can vary. Several factors can influence treatment response, including the stage of the disease, genetic variations, and the presence of comorbidities. The heterogeneity of neurodegenerative diseases and the complex interplay of various underlying mechanisms contribute to the variability in treatment outcomes. It is important to consider these factors when selecting antidiabetic agents and closely monitor patients' responses to treatment. Individualized treatment plans may be necessary to optimize therapeutic benefits [86].

Addressing these challenges requires a personalized approach to treatment, considering each patient's specific needs and characteristics. Close monitoring, regular assessment of treatment response, and vigilant management of potential side effects are essential to ensure the safe and effective use of antidiabetic agents in the context of neurodegenerative diseases. Collaborative efforts between healthcare professionals, researchers, and patients are crucial in navigating these challenges and optimizing treatment outcomes.

Optimal Dosing and Treatment Duration

Optimal dosing and treatment duration of antidiabetic agents for neurodegenerative diseases remain areas of active investigation. Several factors must be considered to determine the most appropriate dosing regimen and treatment duration for individual patients. These factors include the specific antidiabetic agent used, the neurodegenerative disease's stage and severity, the patient's overall health status, and any potential drug interactions [87].

Clinical trials and real-world studies are important for assessing the efficacy and safety of different dosing strategies. These studies help determine the optimal dosage that achieves therapeutic benefits while minimizing the risk of adverse effects. Additionally, long-term studies are needed to evaluate the sustained efficacy of antidiabetic agents and determine the ideal treatment duration [88].

It is important to note that neurodegenerative diseases are chronic and progressive conditions. Therefore, treatment with antidiabetic agents may need to be long-term or lifelong. Regular monitoring of patients, including clinical evaluations and biomarker assessments, can help guide treatment decisions and make adjustments as necessary [89].

Furthermore, individual patient characteristics and preferences should be considered when determining the optimal dosing and treatment duration. Factors such as age, comorbidities, medication adherence, and patient goals should be considered to develop personalized treatment plans [90].

Collaboration between healthcare professionals, researchers, and patients is crucial in optimizing dosing strategies and treatment duration. Ongoing research efforts and clinical trials will continue to provide valuable insights into the optimal use of antidiabetic agents for neurodegenerative diseases, ultimately leading to evidence-based guidelines and individualized treatment approaches [91].

Combining Antidiabetic Agents With Other Therapeutic Approaches

Combining antidiabetic agents with other therapeutic approaches in the treatment of neurodegenerative diseases can potentially offer enhanced neuroprotection and improved clinical outcomes. By targeting multiple pathological processes simultaneously, combination therapies have the potential to exert synergistic effects and provide a more comprehensive approach to disease management [92].

One potential approach is combining antidiabetic agents with medications specifically targeting the underlying pathological features of neurodegenerative diseases. For example, anti-amyloid or anti-tau therapies that aim to reduce the accumulation and aggregation of amyloid-beta plaques or neurofibrillary tangles may be used with antidiabetic agents. The combined effects of reducing protein aggregation and modulating glucose metabolism and insulin signaling pathways could have a greater impact on disease progression [93].

In addition to pharmacological approaches, lifestyle interventions can play a significant role in combination therapy. Lifestyle modifications, such as regular exercise, dietary changes, and cognitive stimulation, have been shown to have neuroprotective effects and can complement the effects of antidiabetic agents. Exercise, for instance, has been associated with improved cognitive function, increased neurotrophic support, and enhanced neuronal plasticity. Combined with antidiabetic agents, these interventions may synergistically promote neuronal health and function [94].

However, it is essential to carefully evaluate the safety and potential interactions of combining antidiabetic agents with other therapeutic approaches. Drug interactions, side effects, and individual patient factors must be considered when designing combination therapies. Rigorous clinical trials and preclinical studies are needed to determine the optimal combinations, dosages, and treatment durations for maximum efficacy and minimal adverse effects [95].

Future research should investigate the efficacy and safety of combination therapies involving antidiabetic agents and other therapeutic approaches. This includes conducting well-designed clinical trials to assess the synergistic effects, long-term benefits, and potential risks of combining different treatment modalities. Furthermore, identifying biomarkers and patient characteristics that can predict treatment response to specific combinations will aid in personalized treatment strategies for individuals with neurodegenerative diseases.

Novel Targets and Emerging Therapies

Glucagon-like peptide-1 receptor (GLP-1R) agonists: GLP-1R agonists, commonly used in managing diabetes, have shown neuroprotective effects in preclinical and early clinical studies. These agents have demonstrated the potential to enhance neuronal survival, reduce neuroinflammation, and improve cognitive function. Further investigations are needed to determine their disease-modifying potential and optimize their use in neurodegenerative diseases [96].

Insulin sensitizer drugs: Novel insulin sensitizer drugs are being developed to target specific pathways involved in glucose metabolism and insulin signaling. By enhancing insulin sensitivity and improving glucose utilization, these drugs may offer improved efficacy and reduced side effects compared to existing antidiabetic agents. Their potential neuroprotective effects and disease-modifying properties warrant further exploration [97].

Neuroinflammation-targeted therapies: Developing therapies that modulate neuroinflammatory responses, such as microglial activation and cytokine signaling, is an active research area. Targeting these pathways may provide more precise and effective treatment options for neurodegenerative diseases. By modulating neuroinflammation, these therapies can potentially mitigate neuronal damage and slow disease progression [98].

Precision medicine approaches: Precision medicine aims to tailor treatment strategies based on an individual's unique characteristics, including genetic profiling and biomarkers. Precision medicine approaches can help identify patients who are more likely to respond to specific treatments or combination therapies in the context of antidiabetic agents for neurodegenerative diseases. By matching patients with the most appropriate therapies, precision medicine holds the potential to enhance treatment outcomes and improve patient care [99].

Continued research efforts and clinical trials are necessary to address these challenges, uncover new targets, and evaluate emerging therapies. Collaborations between researchers, clinicians, and pharmaceutical companies are crucial for advancing the field and translating scientific discoveries into effective treatments for patients with Alzheimer's and Parkinson's diseases. By exploring novel targets and embracing emerging therapies, the field of antidiabetic agents as therapeutic approaches for neurodegenerative diseases can significantly improve patient outcomes and quality of life.

## Conclusions

In conclusion, exploring antidiabetic agents as potential therapeutic approaches for Alzheimer's and Parkinson's diseases holds significant promise. The comprehensive review has provided insights into the potential link between diabetes and neurodegenerative diseases, the shared pathological features, and overlapping molecular pathways. Antidiabetic agents, originally developed for managing diabetes, have demonstrated neuroprotective effects through the regulation of glucose metabolism, anti-inflammatory actions, oxidative stress modulation, and mitochondrial function improvement. In vitro and animal studies have provided compelling evidence of the neuroprotective effects of antidiabetic agents, showcasing improvements in neuropathology and behavioral outcomes. While clinical studies investigating the use of these agents in neurodegenerative diseases have shown mixed results, ongoing research, and larger clinical trials are needed to determine their efficacy, optimal dosing, and long-term safety profiles. Limitations such as drug-specific side effects and variability in treatment response should be addressed to maximize the potential benefits of these agents. Future research should further elucidate the underlying mechanisms through which antidiabetic agents exert their neuroprotective effects. Large-scale clinical trials incorporating diverse patient populations and evaluating long-term outcomes are essential for establishing the effectiveness and safety of these therapeutic approaches. The exploration of combination therapies, precision medicine approaches, and novel targets holds promise for enhancing treatment outcomes in neurodegenerative diseases.
